# *Ex-vivo* Clonally Expanded B Lymphocytes Infiltrating Colorectal Carcinoma Are of Mature Immunophenotype and Produce Functional IgG

**DOI:** 10.1371/journal.pone.0032639

**Published:** 2012-02-29

**Authors:** Claudia Maletzki, Annika Jahnke, Christiane Ostwald, Ernst Klar, Friedrich Prall, Michael Linnebacher

**Affiliations:** 1 Division of Molecular Oncology and Immunotherapy, Department of General Surgery, University of Rostock, Rostock, Germany; 2 Institute of Pathology, University of Rostock, Rostock, Germany; University of Massachusetts Medical School, United States of America

## Abstract

**Background:**

Tumor infiltrating B cells (TiBc) have not yet been investigated in detail. This may at least in part be due to technical difficulties. Here we describe a straightforward and reproducible method to isolate and culture TiBc from primary colorectal carcinomas (CRC).

**Methods/Results:**

TiBc cultures were generated by Epstein-Barr virus (EBV) immortalization. With this method, monoclonal TiBc cultures were obtained for 14/19 CRCs. As assessed by flow cytometry and ELISA, TiBc showed an activated immunophenotype (CD23^+^, CD80^+^) and produced immunoglobulin (Ig; IgG secretion in 55% of the cultures). In functional *in vitro* analysis, most of the IgGs specifically bound to allogeneic CRC target cells. These data suggest that TiBc are antigen-experienced and thus may exhibit functionality *in situ*. Additionally, mini-cultures generated from 12 further CRCs revealed TiBc outgrowth exclusively in the presence of EBV.

**Conclusion:**

In summary, this simple method provides a cellular tool and our data set the stage for analysing the bivalent role of TiBc; being antigen-presenting cells on the one hand and tumor-specific antibody producers on the other. Additionally, the generation of long-term TiBc cultures and their monoclonal Ig may serve to identify novel tumor-specific antigens.

## Introduction

Lymphocytes are major components of mononuclear cells infiltrating human malignant tumors. Their positive prognostic relevance is well established [1]–[3]. Recent studies focused on T cells, for they are believed to have the greatest antitumoral potential [4]. Indeed, a dense infiltrate of CD8+ cells was shown to correlate with prolonged survival of colorectal carcinoma (CRC) patients [5], [6].

However, tumor-infiltrating B cells (TiBc) may play an important role, too. They are considered to be positive regulators of immunity often collaborating with T cells to generate potent, unrelenting immune responses [7]. Beside their antibody (Ab) producing capacity, they enhance T cell responses by secreting stimulatory cytokines and chemokines, or by serving as local antigen-presenting cells (APCs).

The tumor entity in which most efforts have been undertaken in this regard is breast cancer. There, TiBc are present in about 24% of tumors and comprise up to 40% of lymphocytic infiltrates [8], [9]. They have been shown to undergo antigen-driven clonal proliferation and affinity maturation in situ [10]. Very recently, in a large patient cohort of different histological and biological subtypes, Mahmoud and colleagues provided evidence for a favorable outcome when high numbers of CD20^+^ TiBc are present. Of note, this prognostic value was independent from any clinicopathological marker [11]. So far, very few studies assessed the functional relevance of TiBc and the antibodies produced thereof. Using recombinant Ab cloning techniques, Hansen et al. reported an antigen-driven humoral immune response directed against β-actin exposed on apoptotic mammary carcinoma cells [12]. Following engraftment of lung cancer tissue in immunodeficient mice, Yasuda and co-workers identified TiBc producing tumor specific Abs against mutated p53 [13]. Thus, at least a proportion of TiBc accumulating in solid tumors can produce tumor antigen specific antibodies. TiBc could consequently be helpful for the identification of novel or at least of relevant tumor specific antigens (TSA).

Despite the obvious importance of B cells in immunological circuits, the functional role of TiBc was so far not examined in detail. This may be attributable to the difficulties in obtaining sufficient cell numbers, either by fresh isolation or by long-term culture. To overcome this obstacle, we designed a direct and reproducible method to generate immortalized B cell lines from primary tumor tissues. We used EBV-transformation by culturing B cells in the presence of virus-containing supernatant of the marmoset cell line B95/8 [14]. Clonality of outgrowing TiBc cultures, isotypes and function of secreted immunoglobulins (Ig) were analyzed.

## Materials and Methods

### Tumor specimens

Resection specimens of primary CRC without prior chemo- or radiotherapy (n = 19) were received fresh from surgery. Prior written informed consent was obtained from all patients, and all procedures were approved by the Ethics Committee of the University of Rostock (reference number II HV 43/2004) in accordance with generally accepted guidelines for the use of human material.

Molecular classification was done according to [15]. These data as well as staging information compiled from the clinical charts are summarized in Table 1.

**Table 1:** Data of colorectal carcinomas used for generation of TiBc bulk cultures, flow cytometry of CD19^+^CD20^+^ B cells from primary tumors and overall results of outcomes.

Tumor-ID	Age/Gender	Site	TNM-Stage	Molecular type	TiBc culture	B cells in primary CRC [%]
HROC56	70/m	right colon	G1T3N0M0	ND	failure	<0.1
HROC57	43/m	right colon	G3T3N2M1	neuroendocrine	success	0.7
HROC59	76/m	right colon	G2T3N1M1	spStd	success	3.0
HROC60	71/m	right colon	G2T2N0M0	CIMP-H	success	3.0
HROC61	57/m	rectum	G3T3N0M0	spStd	success	NA
HROC62	84/f	right colon	G3T4N2M0	spStd	failure	4.1
HROC63	81/f	left colon	G2T4N0M0	spStd	failure	NA
HROC64	71/m	left colon	G2T2N0M0	spStd	failure	NA
HROC66	75/m	rectum	G2T3N2M0	spStd	success	<0.1
HROC67	54/m	left colon	G2T3N1M0	spStd	success	NA
HROC68	84/m	left colon	G2T4N2M0	spStd	success	NA
HROC69	62/m	right colon	G3T3N0M1	spStd	success	1.1
HROC71	52/m	right colon	G2T3N0M0	HNPCC	†	<0.1
HROC83	85/f	right colon	G2T3N1M1	spStd	†	NA
HROC84	88/f	left colon	G2T3N0M0	spStd	†	0.6
HROC85	65/m	rectum	G2T3N0M0	spStd	success	NA
HROC86	79/f	left colon	G2T3N1M0	spStd	success	<0.1
HROC87	76/f	left colon	G3T3N0M0	spMSI	failure	NA
HROC90	84/f	rectum	G2T2N0M0	ND	success	1.9

NA – not analyzed;

† - no long-term culture established;

ND – not yet determined.

### Tumor cell lines and culture media

CRC cell lines HROC24 (MSI, Kras^wt^, Braf^mut^) and HROC46 (MSS, APC^mut^, Kras^mut^, Braf^wt^), were established in our lab from resection specimen [16]; HCT116 cells were originally obtained from ATCC, Rockville, MD, USA. Cells were maintained in culture medium (DMEM/HamsF12 supplemented with 10% fetal calf serum (FCS), glutamine (2 mmol/l) and antibiotics (penicillin/streptomycin/gentamycin)).

### Generation of TiBc cultures from primary tumors

Tumor fragments were cut into small pieces and homogenized. Cell suspensions were obtained following nylon passage. Either a total of 5×10^6^ cells were cultured in a cell culture flask or cells were distributed in one 48-well plate in the presence or absence of EBV-containing supernatant of B95/8 cells. To prevent T and NK cell growth, cyclosporin A (5.5×10^−7^ M) was added. Typically, outgrowth of TiBc cultures was observed after a period of 4–8 weeks. Established TiBc were harvested, expanded and further characterized. All cell culture reagents were obtained from PAA (Cölbe, Germany), antibiotics and antifungal agents were from the pharmacy of the university hospital Rostock.

### Clonality analysis of TiBc

Genomic DNA was extracted from pelleted TiBc cultures with the Qiagen extraction kit (Hilden, Germany). Clonality was tested using the BIOMED-2 multiplex PCRs protocols to detect Ig gene rearrangements with Genescan detection [17]. Denatured PCR products are size separated in a denaturing capillary sequencing polymer and detected via automated scanning with a laser. Monoclonal cell samples will give rise to PCR products of identical size (single peak), whereas in polyclonal samples many different IGH PCR products will be formed, which show a characteristic Gaussian size distribution.

### Histological and immunohistochemical assessment of TiBc

Peritumoral lymphocytic infiltration patterns were assessed by routine H&E staining. Additionally, representative paraffin-blocks were selected for immunohistochemistry with commercial antibodies directed against CD3, CD20, CD79a, and CD21 using standard protocols.

### Flow cytometric phenotyping

Activation and differentiation status of TiBc were traced by flow cytometry using the following FITC-, PE or APC-conjugated Abs: CD3, CD19, CD20, CD23, CD38, CD27, CD80, MHC I (Immunotools, Friesoythe, Germany), CD83, CD138, and MHC II (Miltenyi Biotec, Bergisch-Gladbach, Germany). Samples were analyzed on a FACSCalibur Cytometer (BD-Biosciences, Heidelberg, Germany). Data analysis was performed using CellQuest software (BD-Biosciences).

### HIg- and Cell-ELISA

Ig isotype production of TiBc was quantified by ELISAs specific for IgG, IgA, IgM, IgD and IgE (Bethyl Laboratories, Montgomery, TX, USA). Briefly, ELISA plates were coated with anti-human Ig (50 µg/ml; 4°C), washed and blocked. TiBc supernatants (1∶50; collected for a period of six month) were added, bound Ig was detected with horse-radish-peroxidase-conjugated anti-human Ig (1∶50.000), and visualized by 3.3.5.5-Tetramethylbenzidin. Reactions were stopped using 2 M H_2_SO_4_. Absorbance was measured on an ELISA plate reader at 450 nm (reference 620 nm). Ig concentrations were determined by comparison with a standard curve generated from serial dilutions of Ig standards.

For Cell-ELISA, 96-well plates, seeded with fixed tumor cells, were used. All washing and blocking steps were done as described. One µg/well IgG was added and incubated at room temperature (2 h). Therapeutic Abs Cetuximab™ (anti-EGFR) and Trastuzumab™ (anti-Her2/neu) served as positive, Rituximab™ (anti-CD20) as negative controls. As a measure for IgG tumor cell binding, extinctions from the background (medium alone, <0.20) were subtracted.

### *In vitro* Binding assays

CRC cell lines (0.5×10^6^) were fixed in 4% formalin for 30 min and incubated with IgG-supernatants from several TiBc cultures (1 µg/ml; 1 h; 4°C). Therapeutic control Abs were applied as described. After washing, cells were stained with secondary anti-human IgG-FITC conjugated Ab (1∶40). Data acquisition was done as described.

Immunofluorescence was performed with fixed tumor cells followed by blocking (2% BSA; 1 h) and incubation with IgG-supernatants from selected TiBc clones (1 µg/ml, 2 h). Afterwards, Alexa 546-conjugated anti-human IgG (1∶100, 1 h) was applied. Cell nuclei were counterstained with DAPI. Tumor cell binding was visualized by fluorescence microscopy (Leica DM 4000 M; Leica Wetzlar, Germany). Pictures were obtained using the provided evaluation software (Leica Image Pro Plus 6.0 image analysis software).

### Statistics

Statistical differences were determined by using the two-sided Fisher's exact T-test. The tests were performed with Sigma-Stat 3.0 (Jandel Corporation, San Rafael, CA, USA). The criterion for significance was taken to be p<0.05.

## Results

### Patterns of lymphocytic infiltration

**Figure 1:**
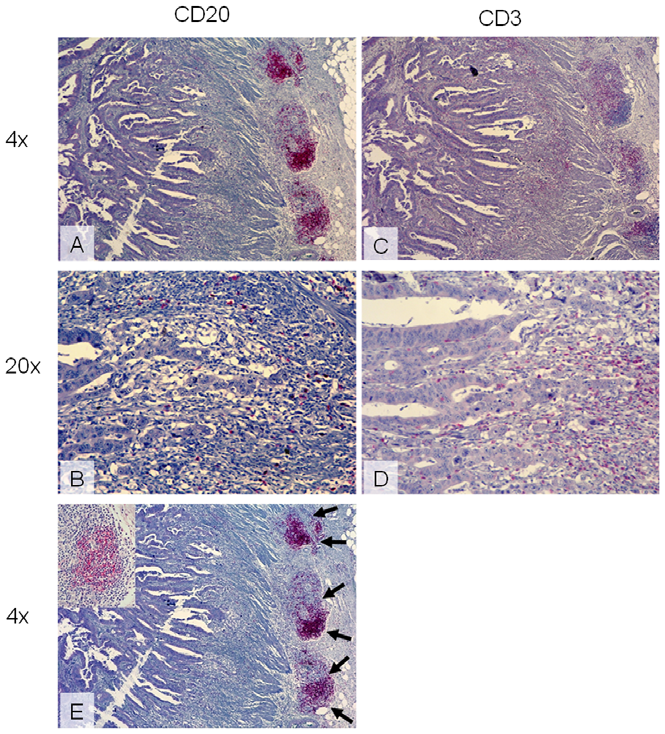
Tumor-infiltrating lymphocytes in a primary colorectal carcinoma (HROC60) highlighted by immunohistochemistry. (A, B) TiBc are observed to concentrate in follicular aggregates at the invasive margin. (C, D) Diffuse T cell infiltration within tumor stroma and to a minor extend within neoplastic glands given by positive staining for CD3. (E) TiBc in follicular aggregates (arrows) reside in a mesh of follicular dendritic cells (inset). Original magnification (A, C, E) 4× and (B, D) 20×.

TiBc were observed to concentrate in the stroma at the invasive margin, and they were never seen between the epithelia of the neoplastic glands (Figure 1A, B). Distribution of TiBc, tumor-infiltrating CD3^+^ T cells, as well as mature plasma cells was quite heterogeneous, even within a single tumor (Figure 1 C, D). In addition, at the invasive margin follicular aggregates of TiBc residing in a mesh of follicular dendritic cells were seen (Figure 1E, inset), and these varied in frequencies between cases.

### Generation of EBV-transformed TiBc cultures

Establishment of TiBc bulk cultures was attempted by EBV transformation for 19 tumors (Table 1). Initially, we generated 14 TiBc bulk cultures (74% success rate) but permanent growth could not be obtained in three cases. However, the remaining 11 cultures proceeded to grow continually. In these, a characteristic *in vitro* pattern of follicular aggregates was observed – similar to peripheral EBV-transformed B cells. Success in obtaining cultures did not correlate with density or pattern of lymphocytic infiltration of the primaries. Moreover, it was quite independent from the amount of TiBc harvested upon initial isolation (Table 1). However, due to the technical challenge of FACS analysis from primary tumor tissue, those data on CD19^+^CD20^+^ B cells in primary CRC need to be interpreted with caution.

To sum up these findings, this method circumvents problems implicated with low B cell numbers obtained by isolation from fresh primary tumors and the technical demands of handling freshly isolated TiBc [11].

### TiBc cultures are of clonal origin

Clonality analysis for IGH and IGK gene rearrangement was performed from the 11 TiBc bulk cultures. Comparison of V(D)J rearrangements and junctional mutation patterns revealed monoclonality in each of the established culture. By applying Gene Scanning fragment-length PCR, predominant clonal rearrangement (monoclonality) was identified as a single peak consisting of one type of PCR product (exemplary results are given in Figure S1A). Analysis of early passages of the cultures demonstrates, however, oligoclonal junctional mutation patterns and thus implies that monoclonality is the result of continuous *in vitro* selection (Figure S1B).

Equally important, demonstration of monoclonality for all of the long-term cultures forms the basis for TiBc characterization and applying Abs produced thereof for functional analysis.

### TiBc are of mature immunophenotype

The most interesting result was the high expression of the B cell maturation marker CD23, suggesting growth after antigenic stimulus (Table 2). Consistent with ongoing maturation, in each clone a heterogeneous pattern of B and plasma cell markers was observed (Table 2). Expression of the general activation marker CD83 ranged from zero to 60%. None of the clones expressed T cell markers confirming their B lymphoid origin. Expression of MHC class I and II molecules as well as the co-stimulatory adhesion molecule CD80 was very high.

**Table 2:** Immunophenotypes of TiBc clones by flow-cytometry and results of Cell ELISAs.

ID of primaryCRC	Immunophenotype [% positive cells]									Ig-type	Cell ELISA [Ex 495 nm]		
	CD19	CD20	CD23	CD27	CD80	CD83	CD138	MHC I	MHC II		HROC24	HROC46	HCT116
**HROC57**	57	17	90	0	78	23	46	98	100	IgG	0.55	0.44	0.20
**HROC59**	14	17	95	17	75	0	13	94	98	IgG	<0.1	<0.1	NA
**HROC60**	24	3	44	8	48	22	22	97	100	IgM	NA	NA	NA
**HROC61**	33	20	65	2	34	19	19	100	100	IgM	NA	NA	NA
**HROC66**	50	9	89	5	70	1	49	93	100	IgG	<0.1	<0.1	0.50
**HROC67**	57	17	90	0	78	21	46	98	100	IgM	NA	NA	NA
**HROC68**	35	5	92	1	46	8	65	94	100	IgG	0.28	0.37	0.67
**HROC69**	56	39	96	17	22	21	51	99	100	IgA	NA	NA	NA
**HROC85**	64	20	80	0	86	59	11	98	100	IgM	NA	NA	NA
**HROC86**	NA	37	40	38	31	0	55	52	99	IgG	NA	NA	NA
**HROC90**	91	26	53	1	84	35	48	93	100	IgG	0.39	0.53	0.89
										Cetuximab	0.40	<0.1	0.26
										Trastuzumab	0.33	0.15	0.50
										Rituximab	<0.1	<0.1	<0.1

Flow cytometric analysis of established EBV-transformed TiBc cultures (after four to five month in culture). ELISA was performed with the use of supernatants from 11 TiBc clones to determine their hIg isotypes (monitored for a period of six months). NA – not analyzed. Results show data from one representative experiment each performed at least three times in triplicates.

### Ig repertoire produced by TiBc clones

All of the analyzed supernatants contained detectable, though varying levels of either one of the three main Ig isotypes: IgG, IgM or IgA (Table 2). Production of IgD or IgE was not observed in any of the cultures. The pattern of Ig secretion did not alter during the observation period. Importantly, among the 11 cultures that had been included into the analysis, exclusive production of IgG was found in 55% (6/11 cultures).

### IgGs specifically bind to tumor cells

**Figure 2:**
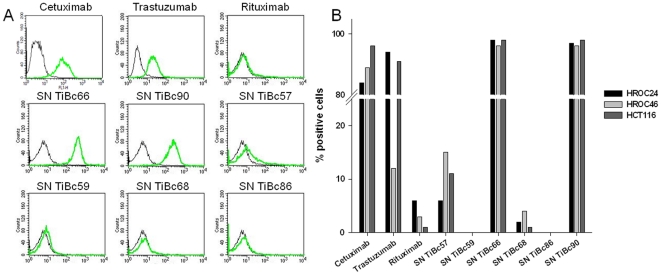
*In vitro* binding assay. (A) Histograms show HCT116 tumor cells following incubation in secondary FITC-conjugated Ab only (black curves), therapeutic Abs (green curves, upper panel) or TiBc-derived IgGs (green curves middle and lower panel). Representative experiments each performed in triplicates are shown. (B) Quantitative analysis for flow cytometric assessment of TiBc-derived IgG binding capacity towards tumor cell targets (HROC24, HROC46, and HCT116). As positive control, fixed cells were stained with therapeutic Abs Cetuximab and Trastuzumab. Negative controls consisted of fixed tumor cells either stained with Rituximab or the secondary FITC-conjugated Ab only.

Of the five TiBc-derived IgGs included into these analyses, four were observed to bind to HCT116 and three to HROC24 and HROC46 (Table 2) in initial cell ELISA screenings. Of note, the affinities of TiBc-produced IgGs were comparable to or even higher than those of the therapeutic Abs specific for EGFR and Her2/neu. These two Abs were chosen as positive controls due to their high expression on the analyzed cell lines (each 80–100%, Figure 2A).

Specific allogeneic tumor cell recognition was confirmed for three of the IgGs by flow cytometry (Figure 2B). IgG from TiBcHROC66 and TiBcHROC90 exhibit strong avidity towards all cell lines tested and both bound to >90% of the tumor cells. Likewise, the mean fluorescence intensity, representing the number of bound Ab molecules per cell, differed only marginally between cells (data not shown). TiBcHROC57-derived IgG displayed an intermediate binding affinity with a maximum of 15% staining for HROC46 cells. When analyzing the other IgGs, weak tumor cell binding if any was observed. Less than 5% of tumor cells allowed binding of IgG from TiBcHROC59, 68, and 86 (Figure 2A).

**Figure 3:**
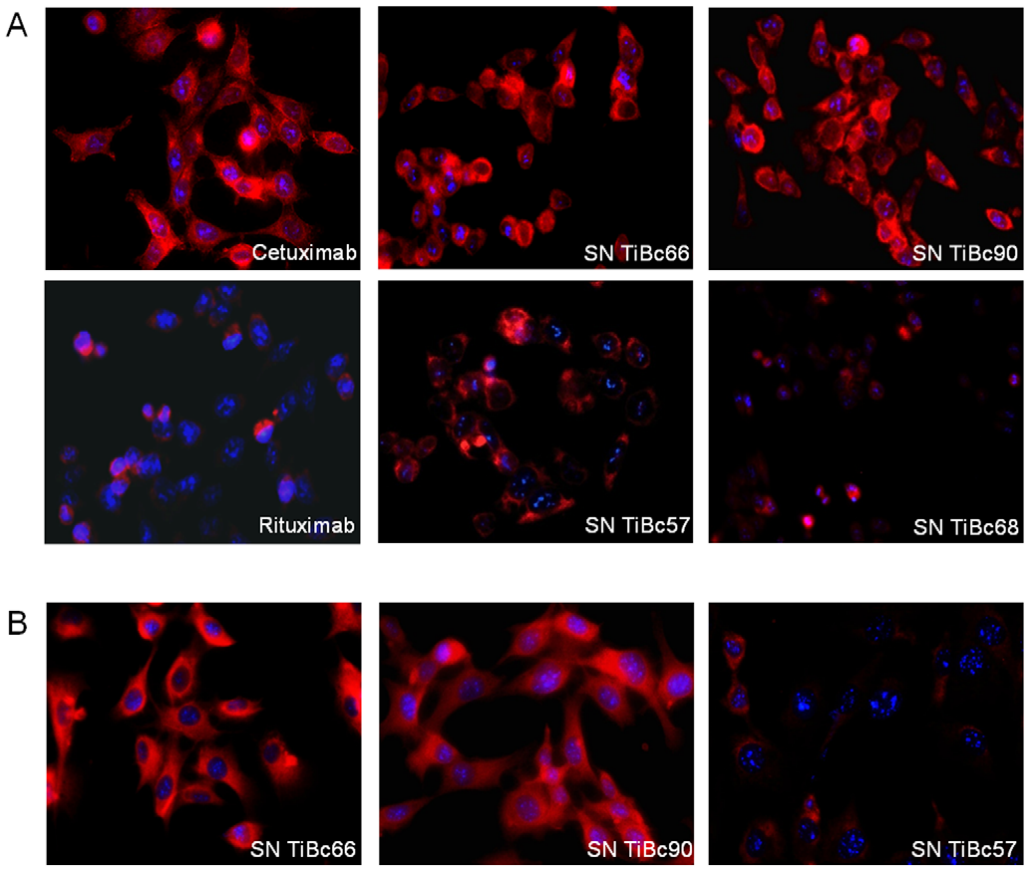
Representative immunofluorescence images of tumor cells showing positive binding of both membranous as well as intracellular target structures by TiBc-derived IgGs. (A) HCT116 and (B) HROC46 tumor cells were stained with TiBc-derived IgGs and Alexa 546-conjugated anti-human IgG. Cell nuclei were counterstained with DAPI. Therapeutic antibodies Cetuximab and Rituximab served as positive and negative control, respectively. Original magnification ×40.

Finally, immunofluorescence studies were done for further confirmation of the above findings and to get an idea of the cellular antigen distribution. Tumor cell recognition was specific, and this was most pronounced for supernatants from TiBcHROC66 and 90. Both IgGs bound to HCT116 cells with high affinity (Figure 3, upper panel). Target cell staining with TiBcHROC57-derived IgG was weaker, but still specific and mostly intracellular (Figure 3, middle panel). Again, negligible staining was shown for TiBcHROC68-derived IgG. Similar observations were made for HROC46 with highest binding for IgG from TiBcHROC66 and TiBcHROC90. TiBcHROC57-derived IgG bound tumor cells to a lesser extent. Positive staining was shown for some, but not all tumor cells (Figure 3, lower panel).

### No evidence for latent EBV-transformation *in vivo*

Growth of lymphoblastoid tumors occasionally complicates xeno-transplantation of tumors in mice [18]. This gave rise to the hypothesis that (some) TiBc could be latently infected by EBV and hide in the immunosuppressive environment of human CRCs to escape EBV-specific T cells [18].

Our *in vitro* system provided a perfect basis to test this hypothesis. TiBc cultures from another 12 CRC were cultured in the presence or absence of EBV (Table 3). There was no outgrowth in any of the cultures without exogenous EBV, whereas with EBV, a total of 97 TiBc clones was obtained from 10/12 cases. Additionally, this simple procedure facilitates the generation of several TiBc clones from one tumor specimen.

**Table 3:** Data of colorectal carcinomas used for generation of TiBc cultures with and without EBV, percentage of CD19^+^CD20^+^ B cells from primary tumors (flow cytometry), and outcomes.

Tumor-No.	Age/Gender	TNM-Stage	B cells in primary CRC [%]	+EBV	−EBV
				No. of clones	No. of clones
1	65/f	G3T4N1	0.6	4	0
2	61/m	G2T3N2	4.4	12***	0
3	69/m	G2T3N0M0	<0.1	9**	0
4	80/f	G2T4N0M0	0.5	10***	0
5	58/m	G2T3N0M0	<0.1	4	0
6	72/m	G2T3N2M1	0.8	11***	0
7	80/m	G2T3N0M1	2.4	9**	0
8	60/m	G2T3N2	0.2	3	0
9	82/m	G2T2N0M0	NA	0	0
10	74/m	T3N2M1	ND	24***	0
11	88/f	G1-G3T3N0M0	<0.1	11***	0
12	78/f	G2T3N2M1	NA	0	0

A total of 5×10^6^ cells were distributed in one 48-well plate in the presence (24 wells) or absence (24 wells) of EBV-containing medium. The cells were maintained in a humidified 37°C incubator with 5% CO_2_. NA – not analyzed. ND – could not be correctly determined.

** p<0.01;

*** p<0.001; Fisher's exact T-test.

## Discussion

In various human cancers, CRC among them, the degree of lymphocytic tumor infiltration correlates with patients' clinical course. Previous studies largely focussed on T cells [5], [6]. Besides, a few preliminary studies described the positive impact on systemic humoral immune responses not only for early detection but also for cancer immunotherapy [19], [20]. We here began to elucidate the role of the humoral anti-tumor immune response within the tumor microenvironment. First, by EBV-transformation we were able to generate long-term monoclonal B-cell lines from TiBc. This procedure was comparatively easy to carry out and quite efficient, yielding 11 lines from 19 CRC specimens. We wish to point out, that this protocol, backed up by proof of monoclonality by the BIOMED protocols used in the diagnostics of lymphomas, offers itself as a basis to start from for any researchers working in this field. It circumvents efficiently the problems implicated in handling fresh TiBc isolates that often are very low in numbers.

The mature immunophenotype of TiBc from primary CRC observed in this study suggests activation and antigen-induced maturation, making accidental tumor tissue infiltration quite unlikely. In agreement with these observations, B cells infiltrating human breast carcinoma tissues were shown to undergo antigen-driven proliferation, somatic hypermutation and affinity maturation, thus representing a tumor specific humoral immune response [11]. This interpretation is further supported by the fact that, as seen by immunohistochemistry, in CRC the great majority of TiBc resides in follicular aggregates. Likewise, peritumoral follicular aggregates of lymphocytes have long ago been reported as “Crohn's like reaction”. This positive prognosticator for CRC was interpreted as an immune-mediated anti-tumor effect [21], [22].

Most of the TiBc cultures started to produce IgG directly after cultivation, IgA secretion was found in one case. Beside IgG, IgA is known to be the other main isotype, secreted exclusively by B cells following a specific stimulus, and this provides a further argument for our hypothesis that B cell infiltration into the tumors is not accidental. Unraveling the functional capacity of this IgA will be of great interest, however in this work we focused on IgG-secreting TiBc cultures. IgG is generally considered to have the greatest potential for therapeutic purposes, because it is most efficient in mediating complement- or NK cell-mediated tumor cell lysis, and has an extended plasma half life [23]–[26].

Using functional assays, specific binding to tumor cells was demonstrated especially for two of the IgGs (from TiBc66 and TiBc90). Both intra- and extracellular antigens were recognized and since all binding experiments were performed in allogeneic settings, these antigens must be shared between different tumor cells. These findings are in line with and add to reports on TSA by other groups [11], [12], [27]. Investigations to identify the antigens bound by TiBc-derived IgGs at the molecular level are on their way, and we are confident that this will help to understand the process of CRC immunoediting [28].

High expression of MHC class I and II molecules as well as the co-stimulatory adhesion molecule CD80 is another interesting finding in this study. This suggests a role for TiBc not only as producers of tumor-specific Abs but also as local APCs, potentially modulating T cell restricted immune responses. One might speculate that tumor-antigen specific TiBc constitute highly efficient APC especially when compared with antigen-unexperienced circulating B cells. However, recruitment of TSA-specific B cells (namely regulatory B cells) by tumor cells in order to suppress antitumoral T cells *in situ* is also imaginable [29].

Finally, it was reported that EBV might induce transient production of low-affinity, unstable IgM [30]. Since isotype switch during long-term culture was not observed in any of the TiBc cultures, we consider it most likely that our cases of TiBc IgM production (n = 4) are a primary antigen-driven immune response and not induced by EBV transformation. It will be of interest to analyze, if stimulation of the IgM-producing TiBc with autologous T helper cells will induce Ig class switching, possibly leading to the production of highly affine IgG. Secondly, since tumor-specific IgM antibodies have been described, specific recognition of tumor structures may be anticipated [31]–[33].

The major findings of our study may be summarized as follows: (a) human CRCs harbor B cells (i.e. TiBc) that can be long-term cultured subsequent to EBV-immortalization; (b) TiBc harvested from these are antigen-experienced and secrete Ig of the three main Ig isotypes (IgG, IgA, IgM) in amounts sufficient for functional studies; (c) IgG derived from several TiBc clones strongly bind to antigenic structures present on several allogeneic tumor cell lines; and (d) TiBc show no evidence for latent EBV-infection *in vivo*.

On the basis of these novel findings, Ig-producing long-term TiBc cultures may provide a convenient cellular tool for the identification of novel or relevant TSA.

## Supporting Information

Figure S1 **Clonality analysis using the BIOMED-2 multiplex PCRs protocols to detect Ig gene rearrangements with Genescan detection.** (A) IGH and IGK gene rearrangement from selected TiBc cultures giving rise to PCR products of identical size (single peak). This confirms monoclonality of TiBc cultures. Monoclonal B-LCLs were taken as control. (B) Exemplary data for early passage TiBcHROC68 culture. Oligoclonality was detected by different PCR products indicating the presence of several subclones at early culture.
(TIF)
