# Supplementary figures and images for: Ex-vivo Clonally Expanded B Lymphocytes Infiltrating Colorectal Carcinoma Are of Mature Immunophenotype and Produce Functional IgG
Source: PLoS One. 2012 Feb 29;7(2):e32639. doi: 10.1371/journal.pone.0032639 (PMC3290587; doi:10.1371/journal.pone.0032639)

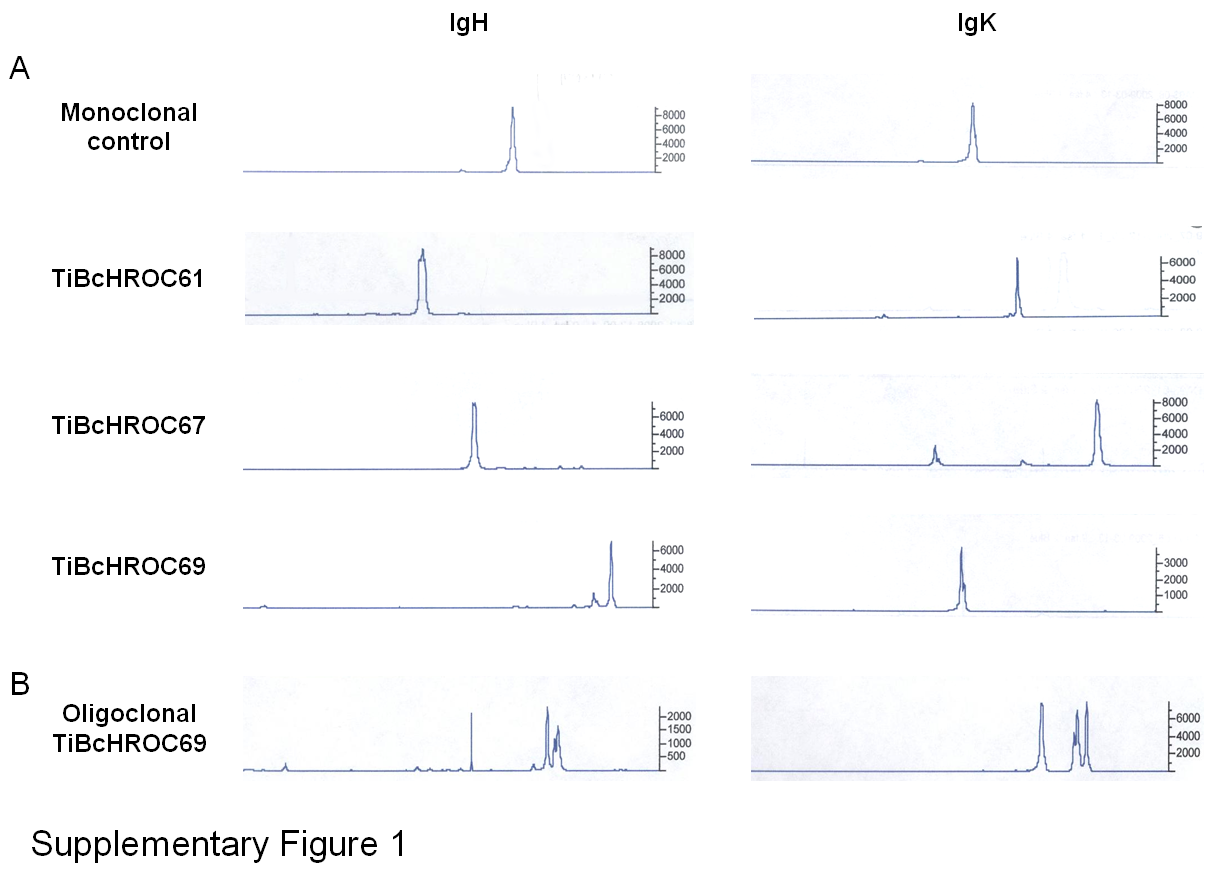

Supplement: Figure S1 — Clonality analysis using the BIOMED-2 multiplex PCRs protocols to detect Ig gene rearrangements with Genescan detection. (A) IGH and IGK gene rearrangement from selected TiBc cultures giving rise to PCR products of identical size (single peak). This confirms monoclonality of TiBc cultures. Monoclonal B-LCLs were taken as control. (B) Exemplary data for early passage TiBcHROC68 culture. Oligoclonality was detected by different PCR products indicating the presence of several subclones at early culture. (TIF) [file pone.0032639.s001.tif]
